# Challenges, benefits, and strategies for delivering pregnancy care to people with disabilities: A qualitative study of service providers and decision-makers in Ontario, Canada

**DOI:** 10.1177/13558196251376146

**Published:** 2025-09-08

**Authors:** Lesley A. Tarasoff, Yona Lunsky, Keat Welsh, Laurie Proulx, Meredith Evans, Susan M. Havercamp, Simone N. Vigod, Hilary K. Brown

**Affiliations:** 1 Department of Health and Society, 33530 University of Toronto Scarborough, Toronto, ON, Canada; 2 Azrieli Adult Neurodevelopmental Centre, 7978 Centre for Addiction & Mental Health, Toronto, ON, Canada; 3 Department of Psychiatry, University of Toronto, Toronto, ON, Canada; 4 Canadian Arthritis Patient Alliance, Ottawa, ON, Canada; 5 Nisonger Center, The Ohio State University, Columbus, OH, USA; 6 7985 Women’s College Hospital, Toronto, ON, Canada; 7 Dalla Lana School of Public Health, University of Toronto, Toronto, ON, Canada

**Keywords:** disability, pregnancy care, policy

## Abstract

**Objectives:**

To (1) understand the challenges and benefits of providing pregnancy care to people with disabilities and (2) identify strategies to address challenges, from the perspectives of health care and social service providers and decision-makers.

**Methods:**

We undertook a qualitative descriptive study in Ontario, Canada, of 31 health care and social service providers and decision-makers. Participants completed semi-structured interviews about their education, training, and clinical or administrative experience working with pregnant and/or parenting people with physical, sensory, and intellectual or developmental disabilities, including challenges and benefits in pregnancy care provision, programming, and policies, as well as their recommendations to improve care. We took a directed content analysis approach.

**Results:**

Participants identified challenges in providing pregnancy care to people with disabilities, including a lack of data to inform care, the influence of social determinants of health on disabled people’s lives, inadequate infrastructure, poor coordination and communication across services, minimal disability-related training, and ableist attitudes among providers. Benefits to providing pregnancy care for people with disabilities included becoming advocates for system-level change, personal fulfillment, opportunities to confront one’s own biases, and development of humility related to the expertise of people with disabilities. Reflecting on these challenges and benefits, participants identified strategies for improving care, through creative resource-sharing solutions, accessibility measures, interprofessional and coordinated care, enhanced provider training, and respectful care approaches.

**Conclusions:**

Findings show the need for changes at system, institutional, and service provider levels to improve pregnancy care for people with disabilities.

## Introduction

Pregnancy rates among people with disabilities in North America have increased over the last 20 years,^1,2^ with a recent study in Ontario, Canada suggesting one in every eight pregnancies are to a person with a disability.^2^ Yet, despite representing a significant proportion of the obstetric population, people with disabilities continue to experience perinatal health disparities and barriers accessing care. Population-based studies show rates of severe maternal morbidity and mortality are higher in people with disabilities than those without disabilities.^3^ People with sensory and intellectual or developmental disabilities also receive prenatal care later than advised and are less likely to receive the recommended number of visits.^4,5^ Qualitative studies have documented obstacles accessing pregnancy-related care in this population, including insufficient provider knowledge and ableism (i.e., social prejudice and discrimination toward people with disabilities based on the assumption that people without disabilities are “superior”), physically inaccessible care spaces, communication barriers, and lack of tailored resources.^6–8^

Less is known about the experiences of health care and social service providers and decision-makers in organising and delivering pregnancy care for people with disabilities. Emerging studies suggest the presence of system-level barriers, including a lack of provider training and education related to disability, few clinical guidelines to support delivery of care, and time and/or resource constraints.^9–17^ Most of these studies are from the US,^9–13^ and they have largely included obstetric providers (e.g., obstetricians, midwives),^9,10,12,13,15,16^ targeted specific disability groups (e.g., people with physical^9–11,16^ or intellectual and developmental disabilities^12,13,15^), and focused on identifying barriers to care, not recommendations for improvements.^9,10,13–15^ Data focusing on how to improve care are needed from diverse health systems, with a breadth of perspectives (e.g., allied health professionals, social service providers, health care administrators) and using a cross-disability approach to identify common considerations across disability groups. Such data can support provider and system change to improve pregnancy care.

We sought to (1) understand the challenges and benefits of providing pregnancy care to people with disabilities and (2) identify strategies to address challenges, from the perspectives of health care and social service providers as well as decision-makers, in Ontario, Canada.

## Methods

### Study overview

As part of a multi-method project examining the pregnancy care experiences of people with physical, sensory, and intellectual or developmental disabilities in Ontario, Canada,^2,3,5,8,17–19^ we conducted a qualitative descriptive study^20^ following the COnsolidated criteria for REporting Qualitative (COREQ) guidelines.^21^ This was a community-engaged study with a 30-person advisory committee comprised of people with disabilities (including several with lived experience of pregnancy), disability organization staff, health care providers, and decision-makers, who met regularly to provide input on data collection and analysis and assist with knowledge sharing. The study received approval from the University of Toronto Research Ethics Board (#35018).

### Study setting

Ontario is Canada’s largest province, with 140,000 births per year. Pregnancy-related care with a physician or midwife is provided to residents at no direct cost. Most obstetricians and family physicians are remunerated by a fee-for-service model, wherein they are paid a fee from the Ontario Health Insurance Plan for each service they provide to each patient.^22^ Their expenses (e.g., staff, equipment) come from this payment. Midwives, who manage 20% of births in Ontario, are paid a lump sum for the entire course of perinatal care provided per patient.^23^ Patients can receive their pregnancy care from an obstetrician, family physician, or midwife, and transfers of care can occur (e.g., to obstetricians) during pregnancy due to complications.

Canada ratified the United Nations Convention on the Rights of Persons with Disabilities in 2010. However, disability legislation for accessibility in public spheres (e.g., the Accessible Canada Act^24^ and Accessibility for Ontarians with Disabilities Act^25^), does not currently cover health care. This means there is considerable variation across the province in health care accessibility, and varying levels of knowledge amongst health care providers of their responsibilities.

### Participants

We used purposeful sampling to recruit providers and decision-makers, through direct approach by email or via participants responding to study advertisements shared by professional organization listservs and websites. Participants had to be (1) health care or social service providers in Ontario who had clinical experience working with pregnant and/or parenting people with disabilities, or (2) decision-makers in pregnancy care or disability services at the organisational, municipal, provincial or federal level (e.g., management, leadership, and policy-maker roles). We aimed to include individuals working in a variety of settings (e.g., urban/rural, community/hospital), from a range of disciplines (e.g., obstetrics, midwifery, public health, social work), and with a variety of disability-related expertise (e.g., physical, sensory, intellectual or developmental).

### Data collection

Interested participants were screened against the inclusion criteria above. Participants gave informed consent. We conducted 28 interviews with 31 providers and decision-makers between October 2019 and February 2020 (3 decision-makers representing the same government branch were interviewed together, and two nurse practitioners from the same primary care practice were interviewed together). The interviews were conducted by a postdoctoral fellow with expertise in qualitative research and disability and pregnancy-related health (L.A.T.); five interviews were conducted with a peer researcher with physical disabilities (K.W.). Interviews were conducted via Zoom (*n* = 11), by telephone (*n* = 10), or in-person (*n* = 7). Interviews followed a semi-structured guide that was developed in collaboration with the advisory committee (Online Supplement 1) and queried participants’ training and clinical/administrative experience working with pregnant and/or parenting people with disabilities, including challenges and benefits in care provision, programming, and policy. Participants were also asked for recommendations to improve pregnancy care. Data saturation was determined to be satisfied when consistent themes (e.g., challenges in care provision) were reflected in the responses.^26^

Interviews were audio-recorded and ranged from 11 to 62 min (average 33 min). Interviews with decision-makers (average 26 min) were shorter than those with providers (average 37 min). Upon interview completion, participants were offered a $25 CAD gift card. No transcript or summary of findings was sent to the participants for their correction or comment.

### Data analysis

Immediately after each interview, the interviewers wrote fieldnotes summarising key points to inform recruitment and subsequent interviews (e.g., areas to probe further). Interviews were transcribed *intelligent verbatim* (with removal of redundant sounds and words) and were verified and deidentified by L.A.T.

We used directed content analysis,^27^ guided by a socioecological model to consider factors that influence health care outcomes at systemic, institutional, interpersonal, and individual levels.^28^ To enhance credibility and dependability of findings, we engaged in analyst triangulation.^18^ First, L.A.T. (also the lead interviewer) and M.E. (a medical anthropologist who was not involved in data collection) independently read four transcripts and discussed them to make an initial list of codes. Second, all authors independently read and discussed two transcripts. From this discussion, L.A.T. created the coding framework. Ultimately, the coding framework included inductive and deductive codes, that is, codes derived from reading and discussing the transcripts and codes informed by related studies.^18^ The coding framework was constructed in NVivo 12 Plus, wherein all data were coded by L.A.T. Memos, focused on challenges, benefits, and strategies in pregnancy care for people with disabilities, were generated by the L.A.T., revised by H.K.B. and M.E., and shared with the full author team to create the final set of themes.

### Participants

Participants included health and social service providers (*n* = 20), including physicians, midwives, nurses, and social workers, and decision-makers (*n* = 11), such as provincial policy representatives and organizational leaders (Table 1; Table S2). Many participants were involved in programmes and policies that prioritised care for groups “designated as ‘high risk’ or in need of extra support,” including people with low socioeconomic status, Black and Indigenous Peoples, and young adults. When asked to estimate the percentage of disabled patients in their care settings, most providers estimated less than 25% or noted these data were not collected. However, some providers primarily served people with disabilities, with some participants reporting working with people with a variety of disabilities and others focusing on physical or intellectual and developmental disabilities in their clinical practice. Most decision-makers oversaw programmes focused on pregnancy and child health, and few had disability-related expertise. About half of all participants reported being in their position for more than 10 years. Most worked in urban centres and identified as women. Seven participants identified as having a disability.

**Table 1.** Participants quoted in text.

Reference	Provider/decision maker perspective	Role
P1	Provider	Maternal-fetal medicine specialist
P2	Provider	Obstetrician-gynecologist
P3	Provider	Obstetrician-gynecologist
P4	Provider	Family physician
P5	Provider	Family physician
P6	Provider	Midwife
P7	Provider	Midwife
P8	Provider	Midwife
P9	Provider	Nurse practitioner
P10	Provider	Primary care nurse practitioner
P11	Provider	Advanced practice nurse
P12	Provider	Registered nurse (community)
P13	Provider	Registered nurse (antenatal)
P14	Provider	Registered nurse
P15	Provider	Public health nurse
P16	Provider	Public health nurse
P17	Provider	Developmental social worker
P18	Provider	Registered social worker
P19	Provider	Registered social worker
P20	Provider	Clinician-scientist
D1	Decision-maker	Senior policy analyst (federal government)
D2	Decision-maker	Senior epidemiologist (federal government)
D3	Decision-maker	Senior policy advisor (provincial government)
D4	Decision-maker	Senior policy analyst (provincial government)
D5	Decision-maker	Senior policy analyst (provincial government)
D6	Decision-maker	Director (provincial government)
D7	Decision-maker	Manager (provincial government)
D8	Decision-maker	Program director (disability organization)
D9	Decision-maker	Director (child welfare)
D10	Decision-maker	Director (child welfare)
D11	Decision-maker	Director (child welfare)

### Positionality and reflexivity

Co-authors included people with lived experience of disability and pregnancy, as well as health care research and clinical expertise. In qualitative descriptive research, staying close to participants’ accounts is important.^18^ However, we acknowledge that data analysis inevitably involves interpretation; our personal and professional experiences likely sensitized us to particular aspects of participants’ accounts, including challenges and benefits of care, which may have influenced our construction of themes. Recognizing trustworthiness as an indicator of rigour in qualitative descriptive research,^18^ we engaged in reflexive processes throughout the research, including team discussions about our positionalities and how our assumptions or identities shaped data collection, analysis, and interpretation.

## Findings

**Figure 1.**
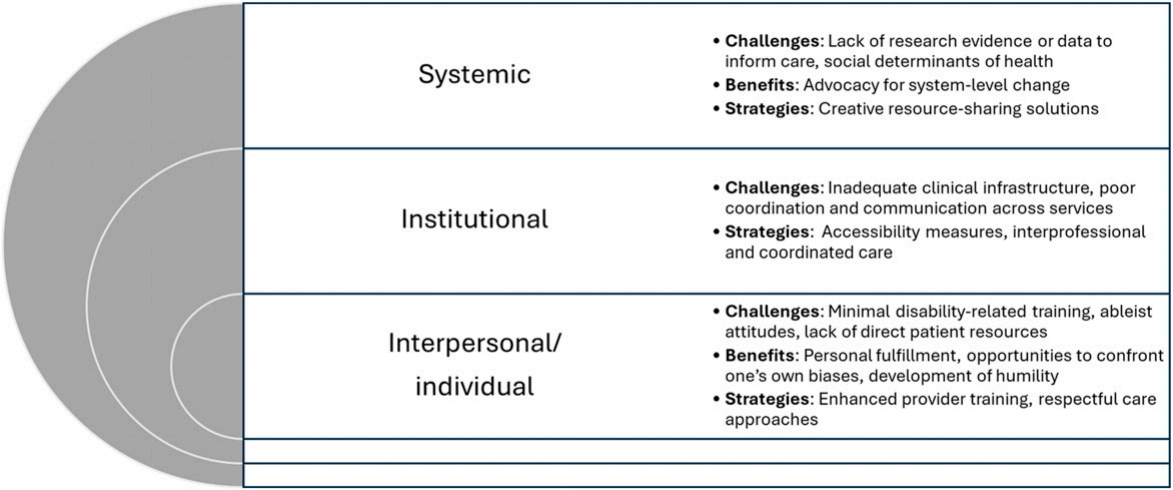
Challenges, benefits, and strategies for pregnancy care for people with disabilities identified by service providers and decision-makers.

Challenges, benefits, and strategies related to pregnancy care for people with disabilities operated on systemic, institutional, interpersonal, and individual levels, summarized in Figure 1 and reported on below.

### Challenges

#### Lack of research evidence or data to inform care

Participants identified a lack of data to inform delivery of pregnancy care to people with disabilities. For health care and social service providers, this impacted their ability to provide evidence-based care. A maternal-fetal medicine specialist noted:

We have to dig. When a lot of [information] is not necessarily published in general obstetric journals, sometimes there’s little snippets that you can grab from a urology journal, a rehab journal, a neurology [journal]… But [disability and pregnancy is] not the focus […] [Information related to different types of disabilities is] all kind of lumped together and, we’re kind of making assumptions […] It’s hard to say, ‘Aha! This applies to this person.’ [P1].

For decision-makers, this lack of data made it difficult to monitor health outcomes and quality of care. As described by a provincial policymaker, “It’s hard to set a baseline for [health outcomes] that we never thought to capture in the first place” [D6].

#### Social determinants of health

Participants also recognized that the ways in which many people with disabilities are marginalized, through experiences of racism and low socioeconomic status, impact how they are perceived and care for. As one social worker described:

I think it’s largely systemic. One of the things I’ve noticed a lot […] is that the number one label attached to our families and moms is ‘disability.’ And then there’s a lack of acknowledging or addressing the other identities that often have more of an impact on their day-to-day lives […] because they already have so many other things that they can’t really hide; their skin colour, their socioeconomic [status]. [P18].

Histories of trauma also made providing care difficult. A social worker noted that:

…many of the women have really extensive trauma backgrounds. Just traumatic experiences with intimate partner violence, sexual violence […] undiagnosed mental health that’s present. [P18]

Some providers cited examples of how colleagues’ assumptions about disabled peoples’ capacity for parenting were shaped by intersecting forms of oppression. One midwife described:

[We had a] client who was Deaf, was older, was married, had finances […] There were no issues regarding child protection or her ability to care for the [child] because of her disability […] However, my client, who was blind and had a mild cognitive impairment, who I felt was very capable of taking care of her child, but was young and was single and was living in significant poverty […] that starts all sorts of child protection concerns. [P6]

#### Inadequate clinical infrastructure

Participants described a lack of infrastructure to support delivery of care. This included a lack of time, which was felt to be due to Ontario’s fee-for-service system which does not provide additional funding for the time that may be needed to accommodate patients with complex needs. Physicians described how “there’s paperwork, there’s phone calls, there’s extra stuff” (family physician [P5]) for disabled patients that require “a little bit more time, a little bit more work, a little bit more creativity” (obstetrician [P3]), and that the lack of remuneration meant “providers are not likely to want to take on any patient that they see as complex” (provincial policymaker [D6]). As one obstetrician shared:

There’s a pie. It’s this big and you don’t suddenly get extra time […] So, I see the patient, it’s taking up all the time, I can’t see anyone else during that time, and I get paid $45, out of which I also have to pay my assistant’s time and everything else. It’s a bit of *pro bono* work […] It’s not a five-minute assessment; it’s a lot longer than that. [P2]

Providers also spoke about inadequate physical infrastructure. Several noted that while they were aware of their responsibility to make their clinics accessible, it was difficult because “it can mean a lot of retrofitting” (obstetrician [P2]). This was the case even in “a brand-new hospital […] Most of the rooms aren’t wheelchair friendly” (clinician-scientist [P20]). One obstetrician who had tried to make their clinic accessible felt a lack of institutional support for their efforts:

We designed [the clinic] so that the hallways and the doorways were appropriate for [electric wheelchairs]. We have one larger exam room with a table that is electric and can act as an exam table that can go right down […] I do all of this provided through my own funding […] At the hospital, really no support. It’s just, I’m given the clinic space. [P3]

Others described a lack of resources to make communication accessible. A primary care nurse practitioner [P9] noted how there was no braille on the signage of their hospital and they were unaware of resources for people with hearing impairments other than speaking clearly to enable lip-reading.

#### Poor coordination and communication across services

Participants identified challenges in coordination and communication between care providers. This sometimes related to coordination of disability-related supports required for some patients to be able to attend their appointments. One obstetrician said:

I know how much time it takes my assistant to coordinate somebody with other supports. It takes her over an hour and a half to coordinate each of these appointments […] They often will need a support person, can that person make it? Have we arranged the para-transport? [P2]

Difficulties also related to the coordination of different medical specialists. A maternal-fetal medicine specialist noted:

Some maternity care hospitals, they only do ‘women’s and babies’ care. They don’t have the other disciplines, which actually irks me because I feel it’s like, ‘Oh, you’re a woman who’s having a baby’ […] [but] she has lots of other organs in her body and […] she’s a full human being, you know? […] I think it takes away a little bit of the ability to provide interdisciplinary care to a woman that might have underlying health concerns. [P1]

#### Minimal disability-related training

A striking challenge was a lack of disability training for providers. An obstetrician noted differences between her training as an occupational therapist and her medical training:

Because I was an occupational therapist […] I spent a lot of time working with clients and families who were caregivers of people with disabilities. And then, as I went into medicine, it was an area that I felt we got no exposure to at all. [P3]

Several providers indicated that disability was noticeably missing in the broader equity, diversity, and inclusion training they received. As one developmental social worker [P17] said, “We have an anti-oppression, anti-racism framework and we receive training probably once every 2 years […] I would say that there’s not a focus at all on disability training in our workplace.”

Others noted the challenge of getting colleagues to be invested in participating in disability-related continuing education, noting this as “a willingness or enthusiasm challenge” (provincial policymaker [D6]). A midwife [P7] praised a birth centre for having a training session presented by a disability organization, but noted that “a lot of midwives didn’t come.” This lack of training was acknowledged to lead to inadequate clinical skills. An antenatal nurse [P13] recalled a colleague who did not know how to operate a Hoyer lift (a floor lift system that uses a sling to safely transfer people with restricted mobility): “We had a new nurse who put a patient up into a Hoyer lift and the lift froze, and the patient was up in the air for 20 min […] Nobody said, ‘I’m sorry, that we didn’t have the right skills.’”

#### Ableist attitudes

Participants acknowledged the negative attitudes held by many of their colleagues about disability and pregnancy, describing this challenge as “the big one […] *we* are the biggest barrier” (midwife [P8]). They described how these attitudes could impact care. An obstetrician noted:

It’s little things. It’s like, [sighs] ‘I don’t know how we’re going to be able to get this patient over the bed.’ Well, you know what? That’s not the patient’s fault […] We shouldn’t be rolling our eyes and making the patient feel badly or sighing because all it takes is an eye roll or a sigh for someone to feel like they’re not important. [P2]

Several providers described colleagues questioning patients’ future parenting abilities. An advanced practice nurse [P11] recalled, “We had a mum who had a healthy pregnancy, she delivered full-term, everything went smoothly […] but [she] was challenged by a staff in the postpartum unit as to, ‘how are you going to take care of your baby if you can’t pick her up?’” Some participants identified instances of colleagues who called child welfare after the birth of a baby to a disabled parent “even if we have successfully advocated with different care providers throughout the pregnancy” (midwife [P8]). Usually, there was no evidence supporting these calls. As described by a child welfare director [D10], “it’s just a suspicion.”

### Benefits

#### Advocacy for system-level change

Several providers described how their work had prompted them to advocate for system-level change. This advocacy was often done on their own time, upon recognizing a “gap in care” and was not “like a billable service” (maternal-fetal medicine specialist) [P1]. An obstetrician described the importance of this advocacy:

[Doing this work] really has led to me to want to advocate for [pregnant people with disabilities] […] that they’re treated with respect, and we have the right equipment for them and we’ve got the people so that they feel safe […] I have the ability sometimes to hugely impact their quality of life. [P2]

#### Personal fulfillment

Health care and social service providers also cited examples of personal fulfillment in providing pregnancy care to people with disabilities. This was felt in the opportunity to see families grow. A social worker [P18] described, “It’s meaningful and rewarding because you’re involved in the most intimate parts of somebody’s life, which is their family and their children.” Others described the satisfaction of supporting everyone’s right to parent. A social worker who worked in a perinatal setting [P19] felt privileged they were able to see “there are possibilities for everyone.”

Several providers also found fulfillment in challenging themselves professionally when they were confronted with the limits to their own knowledge. A maternal-fetal medicine specialist [P1] said: “It’s intellectually very interesting. I like to read a lot. There’s always this continued education; there’s always new things to learn and do.”

#### Opportunities to confront one’s own biases

Participants found working with pregnant people with disabilities caused them to confront their own biases. A family physician described a shift in her thinking from focusing on contraception in patients with intellectual disabilities to supporting their reproductive rights:

I used to feel […] I was a failure if a young woman with [an intellectual disability] came in and was pregnant. Having given her contraception, she either wasn’t taking [it] or wasn’t taking [it] properly. But I’ve realized that’s my own bias and, in several cases, it was very positive that she became pregnant, and she wanted to. [P4]

Those who worked with patients with physical disabilities noted that they had to change their assumptions about who could have a healthy pregnancy. An advanced practice nurse stated:

You see this woman having a healthy pregnancy and having these beautiful babies […] I don’t want to be – I’m surprised, but I am very happy they have such a good outcome and that I’m open to this possibility that everybody with physical disabilities are able to make these choices and they are generally safe choices and have a good outcome. [P11]

#### Development of humility

Several participants described how working with patients with disabilities helped them to develop humility in their work and understand that patients are experts in their own care. One midwife [P8] described, “I’ve learned some acute humility about the expert sitting in front of me. And that I have to acknowledge that I am learning on the backs of their struggle.”

For some providers, this humility meant changing the way they delivered care. One community nurse described realizing she needed to change her communication style to better meet the needs of her clients with intellectual and developmental disabilities:

The experience I had with [patients with intellectual disabilities] was quite profound, because as much as you think you are really providing basic information [in prenatal education classes], I realized I wasn’t at their level, and so that taught me a lot. To really go back and evaluate how I was presenting and ways that I could make it more meaningful and more impactful. [P12].

### Strategies

#### Creative resource-sharing solutions

Participants identified creative resource-sharing solutions. In response to concerns that people with disabilities represent a relatively small proportion of the obstetric population, one provincial policymaker [D4] suggested a “hub and spoke” model, with “a hub somewhere where they’re doing this really well, can we gather some spokes around the province that they can sort of ‘up’ skill, and share information and become a little bit of a centre of that work?” Similarly, a disability program director described the value of a central repository of resources:

It’s a simple thing but nobody has thought about how to put those [booklets] that people can just [pick up one and say] ‘If I need this help, where can I go? If I need a caregiver or I need a clinician, if I need a nurse practitioner, I need a midwife […] I need some assistive devices to take care of my child, where should I go?’ [D8]

#### Accessibility measures

Many health care and social service providers described specific changes to make care accessible. They described the need to change physical spaces, but there was a recognition that “we need to get past that notion that disability just means you’re in a wheelchair” (child welfare director [D10]). A maternal-fetal medicine specialist further described:

[Making pregnancy care accessible means asking] how do we incorporate the woman and her partner into their birth plan? How do we make sure that we’ve positioned people in a way that’s really comfortable for them during birth? Are we communicating well with each other? […] We need some of that [accessible] equipment but it’s not like, ‘We have a scale. Now, we’re an accessible clinic.’ [P1]

Participants noted that accessible care requires time, in the form of longer or more frequent prenatal care appointments. As a midwife [P6] explained, “I’d say book extra time for the visits just so that you’re not rushed, and you can take the time that you need.” A family physician noted how their practice setting might make such flexibility easier than for specialists:

Family doctors are more able than obstetricians to give the time. I try to see them every 2 weeks instead of every 4 weeks, because I find there’s enough to talk about. It helps me keep an eye on them, make sure they’re doing okay and gives them a chance again to touch base, if they’ve got questions or concerns. [P4]

#### Interprofessional and coordinated care

Several providers highlighted the value of interprofessional care for patients with disabilities. A maternal-fetal medicine specialist shared the role of multiple medical specialists:

I have my ‘league of superheroes,’ […] With pregnancy, we don’t have long timescales. I can’t go, ‘I’ll take care of it in a year and a half.’ It doesn’t happen, the baby’s growing, the baby’s coming. We have these short time frames, so it’s nice to know I have this really reliable, wonderful group of people. [P1]

For other patients, providers noted that interprofessional care related to considering not just the individual’s medical needs but also their social needs. One family physician described:

Maternity care is not about just checking on the heartbeat, blood pressure, they’re fine and they’re out the door. I think you need to think about the whole person, about their social situation, what’s happening. [P4]

Such interprofessional care was recognized by participants to require communication and coordination across providers and sectors. A developmental social worker noted:

There needs to be much broader organization and communication within agencies. Some of our biggest fights happen [when] agencies […] are declining to support families and [have] excuses, ‘oh the Ministry needs X, Y and Z to happen.’ So many of the systems, they demand that parents jump through unreasonable hoops to access their services. [P17]

As a practical example of coordination, a maternal-fetal medicine specialist [P1] described the advantage of having appointments with different specialists on one day to reduce the burden on the patient: “We consolidate it, so they don’t need to come on Tuesday, see the dietician, and then see their neurologist on the Wednesday and then come see me and have their scan on Friday.” Recognizing the complexity of coordinating different services and care providers, an obstetrician [P2] suggested, “you need a coordinator for these things,” emphasizing the valuable role of patient navigators. An advanced practice nurse [P11] often acted in this coordination role, acting as a central point of contact for patients who were otherwise seeing several different specialists; she noted that: “the patients are mostly assigned to me in that we can kind of follow that patient throughout their pregnancy.”

#### Enhanced provider training

Many participants advocated for training for providers, noting that “if somebody wants to be working with somebody within the disabled or d/Deaf community, I think they need to demonstrate they’ve done some training” (midwife [P8]). Participants described how such training must include interaction with people with disabilities, since “lived experience is key” (disability programme director [D8]). A family physician [P4] described an innovative family medicine residency programme where residents were paired with patients with intellectual and developmental disabilities and followed their care for a year, noting that “it’s mutually beneficial.” Several participants also indicated that training needs to include development of “skilled language on asking clients what they need” and the need to “challenge expectations and assumptions about who is a good or fit parent” (midwife [P6]).

Others noted it might not be realistic to expect that everyone has specialised disability training, but that broader awareness would be beneficial. A midwife [P6] suggested, “I think some demystification on a really broad level would be great […] You don’t need to be an ‘expert’ on disability in pregnancy but here are some key things that you can do to provide good care.”

#### Respectful care approaches

Several providers emphasised the need for respectful care. A public health nurse [P15] noted that “[patients]… are all different and we need to make sure we are promoting messages in a way that will resonate with [everyone] regardless of their situation. Because we want [everyone] to feel comfortable.” Others noted the need for care that emphasises individual autonomy. As described by an obstetrician [P3], “I think that the care has to be provided in a way that allows [patients] to still feel like they have control over their bodies and decision-making over their bodies.” Others similarly noted that such autonomy requires recognition by “people higher up who make policies” (public health nurse [P16]) and by providers that the patient is the expert in their care: “[we have to] listen to the families and take direction from the families […] Too often there’s been this paternalization of the work that happens and this sense of the expertness that comes with [health care provision] and it can’t be that way” (developmental social worker [P17]).

Communication with patients throughout their pregnancy was recognized as a vital aspect of respectful care. As noted by a social worker:

“the cases that I’ve had the most success in, is when we were all communicating, we were meeting regularly with our mom, we were planning together.” [P18]

A maternal-fetal medicine specialist explained how communication is particularly important during critical periods such as the first prenatal care appointment and the birth:

[Advanced practice nurse] will call the person prior to the initial visit to get to know them and make sure that, when they come, it’s a smooth process […] Usually in the mid-third trimester, we’ll have a team meeting where we have all of the individuals who have been involved in that person’s care plus the triage nurse up on the birth unit, the postpartum care team, and the woman and her partner. We have a plan that is written up […] Then the woman and her partner have a copy, so we have, in an emergency situation, this is what the plan is; in a planned situation, this is what the plan is. [P1]

Finally, several providers emphasized how respectful care is particularly important in pregnancy given what a monumental period it is. A community nurse [P12] said, “having a child, that experience is ingrained on your brain forever, either bad or ugly […] it can be so empowering if it’s a good experience.”

## Discussion

In this qualitative study in Ontario, Canada, we identified challenges, benefits, and recommendations to improve pregnancy care for people with disabilities. Service providers and decision-makers described challenges in the organisation and delivery of care, including a lack of research evidence, the complex social circumstances of many patients, inadequate infrastructure, poor coordination and communication across services, minimal provider training, and ableist attitudes. They also identified benefits for service providers and decision-makers of providing pregnancy care for people with disabilities including becoming advocates for change, personal fulfillment, opportunities to confront their own biases, and development of humility about the expertise of patients with disabilities. Reflecting on their experiences, participants identified strategies to improve care, including creative resource-sharing solutions, accessibility measures, interprofessional and coordinated care, enhanced provider training, and respectful care.

Our study affirms the results of prior studies that have identified barriers to the organisation and delivery of pregnancy care for people with disabilities, including a lack of preparedness of the health care system to facilitate accessible care due to the absence of clinical guidelines, insufficient provider training related to disability and pregnancy, and significant constraints on time and resources.^9–17^ Most previous studies were conducted in the US^9–13^ – where health care is paid for by a combination of public programmes, private insurance, and out-of-pocket payments – and focused on a narrow range of providers (e.g., obstetricians)^9,10,12,13,15,16^ and disability types.^12,13,15^ Our research adds to this literature by showing that many of these challenges persist in Canada, which has a universal healthcare system. By adding the perspectives of decision-makers and social service providers, our study also identified system-level challenges (e.g., a lack of research evidence to inform planning) and highlighted the complexity of pregnancy care through both a medical and social services lens (e.g., addressing social determinants of health and issues such as child welfare). Unlike prior studies,^9–13,15,16^ participants were asked to consider the needs of patients with a range of disabilities, including physical, sensory, and intellectual or developmental disabilities. This gave us greater insight into commonalities and differences in experiences for different disabilities. For example, while many providers identified challenges related to infrastructure, the nature of the challenge (e.g., physical infrastructure or resources related to communication) differed by disability type. There were also commonalities in challenges (e.g., lack of data, poor coordination of services) and benefits (e.g., confronting ableist biases) that applied across disabilities. Finally, by asking about recommendations to improve care, we identify strategies from an interprofessional, cross-disability perspective that could make pregnancy care more accessible and inclusive.

Many of the challenges identified in our study relate to the history of ableism entrenched in society and health care systems globally. During much of the 20^th^ century, government-supported institutionalization and involuntary sterilization practices limited childbearing for people with disabilities, informed by eugenic assumptions that disability is a negative characteristic to be prevented.^28^ Although these practices were made illegal in Canada and elsewhere in the 1970s, this history has contributed to persistent ableist societal assumptions about the ability of people with disabilities to parent and their rights to have children.^29^ While the United Nations Convention on the Rights of Persons with Disabilities is intended to protect reproductive rights, health care systems have failed to consider disability in the planning and delivery of pregnancy care.^24,25^ These ableist assumptions and exclusions form the background to a system of pregnancy care that does not prioritize accessibility, leading to the gaps in policy, training, and resources identified by our participants. Yet, resistance in the face of these challenges was recommended by participants as they identified actionable areas for change.

Our study has several limitations. While we aimed to interview participants in a variety of roles with disability-related expertise to generate recommendations, many of the decision-makers felt they had insufficient disability-related expertise, resulting in shorter interviews that lacked the depth of the provider interviews. While we successfully recruited service providers with disability-related expertise, our data may not represent the challenges faced by, and needs of, providers with no knowledge about, or appreciation of, the importance of accessible pregnancy care; their needs could be an area for future research. Most providers with disability expertise worked with people with a variety of disabilities or reported specific expertise in physical or intellectual and developmental disabilities; few participants worked with patients with hearing or vision impairments. Recommendations related to these groups may not be well-reflected in our study. Most participants worked in urban settings, resulting in little data on challenges and strategies pertinent to rural and other under-resourced settings. Finally, our recommendations are gender-inclusive and therefore use the gender-neutral language of “pregnant people.” However, our study did not address any specific questions of gender identity and pregnancy.

Our study has implications for practice and policy in Canada, which are also broadly applicable given the congruence of our findings with prior studies from the US and elsewhere. At a system level, we show the need for resources to support providers’ delivery of pregnancy care to patients with disabilities. This process of equipping providers should start early, through inclusion of disability competency training in medical and social services education that emphasize the development of skills related to the respectful delivery of care to patients with disabilities. For providers, our findings support the need for system-level and institutional infrastructure to facilitate delivery of accessible care. In Ontario, for example, this could be via flexible remuneration systems (e.g., incentive billing codes that allow longer visits for priority populations)^30^ and funding to make health care spaces and communication accessible. Our findings also suggest accessible care could be facilitated through greater cooperation across medical specialists, and between the health and social services and disability advocacy organisations; this will require challenging pregnancy care’s typically siloed approach and require human resources (e.g., patient navigators) to assist coordination. These changes require disability legislation that addresses healthcare standards, including for pregnancy care. Given that patients with disabilities account for 13% of the obstetric population,^2^ these changes are critical to ensure pregnancy care is accessible to all.^30^

## Supplemental Material

Supplemental Material - Challenges, benefits, and strategies for delivering pregnancy care to people with disabilities: A qualitative study of service providers and decision-makers in Ontario, Canada Supplemental Material for Challenges, benefits, and strategies for delivering pregnancy care to people with disabilities: A qualitative study of service providers and decision-makers in Ontario, Canada by Lesley A. Tarasoff, Yona Lunsky, Kate Welsh, Laurie Proulx, Meredith Evans, Susan M. Havercamp, Simone N. Vigod and Hilary K. Brown in Journal of Health Services Research & Policy

## Data Availability

The participants of this study did not consent for their data to be shared publicly, so supporting data are not available.
