# Supplementary material for: Challenges, benefits, and strategies for delivering pregnancy care to people with disabilities: A qualitative study of service providers and decision-makers in Ontario, Canada
Source: J Health Serv Res Policy. 2025 Sep 8;31(1):14–24. doi: 10.1177/13558196251376146 (PMC12647371; doi:10.1177/13558196251376146)
Supplement: Supplemental Material - Challenges, benefits, and strategies for delivering pregnancy care to people with disabilities: A qualitative study of service providers and decision-makers in Ontario, Canada [file sj-pdf-1-hsr-10.1177_13558196251376146.pdf]

Journal of Health Services Research & Policy
Challenges, benefits, and strategies for delivering pregnancy care to people with disabilities: A qualitative study of service providers and decision-makers in Ontario, Canada
Tarasoff L, et al

Online Supplement 1: Interview Guide

1. How did you come to work with women with disabilities/this population?
Prompt examples: How did you come into your current role?
Personal interest/experience?
For service providers: Where did you learn about caring for women with disabilities? Did you receive any training on providing care to this population in [medical, nursing, etc.] school? Did you learn on the job/fly?

2. Can you tell me about the types of services you or your organization provide?
Prompt example: Have services been adapted to women with disabilities?

3. For providers: **Can you tell me about the women with disabilities you typically care for?**
Prompt examples: Physical, sensory, intellectual, and/or developmental.
At what stage of their pregnancies do you typically see them [women with disabilities]?
In your current practice, do you have specialized/assistive equipment in office? If yes, specify what kinds of specialized/assistive equipment are available in your office.

4. For service providers and/or decision-makers representing an organization: **Can you tell me about the women with disabilities that your organization serves or focuses its work on?**
Prompt examples: Physical, sensory, intellectual, and/or developmental.

5. In your experience, are there common complications and challenges women with disabilities experience in the perinatal period?
Prompt examples: Can you please tell me more?
For service providers: In your practice, what are common complications and challenges women with disabilities experience in the perinatal period?

6. If yes to #5: What factors do you think might contribute to or explain the challenges and complications women with disabilities experience during the perinatal period?
Prompt examples: Why do you think women with disabilities are at greater risk of experiencing pregnancy and birth complications? What might be contributing to these perinatal health disparities?

7. Based on your experience, what services or supports are most helpful to women with disabilities during the perinatal period?
Prompt for each stage of the perinatal period.
Is there a specific person, organization, or resource that has been particularly helpful to you in providing care to women with disabilities?
Or what services or supports do you think would be most helpful to women with disabilities who are pregnant and parenting?

8. What are the strengths of current perinatal care services for women with disabilities?

9. What are the gaps or challenges of current perinatal care services for women with disabilities?

Prompt examples: What kinds of resources, knowledge, capacity, or skills do you (as a service provider or decision-maker) need to better address the perinatal health needs of women with disabilities? What do you think might be contributing to or explain these gaps or challenges? What might make it difficult for women with disabilities to get the care they need during the perinatal period?

10. How has working with women with disabilities impacted you or your work?

Prompt examples: Can you recall a specific experience where your views of women with disabilities changed, or you learned something new caring for women with disabilities? What do you wish you knew before working with women with disabilities, or in the area of disability and/or perinatal health?

11. Have you been involved in any educational initiatives on the topic of perinatal health and disability?

Prompt examples: How did these initiatives come about? Who was involved? Were disability organizations or women with disabilities involved? What were the results?
If no, do you have any ideas about interventions to educate service providers, researchers, and/or decision-makers about the perinatal health and health care experiences of women with disabilities?

12. What advice would you give to another person in your position to improve the perinatal health and health care experiences of women with disabilities?

13. What recommendations might you make to improve perinatal care for women with disabilities?

Prompt examples: If you could provide the larger health care system or your local health region or hospital with suggestions to improve care for women with disabilities during the perinatal period, what might these suggestions be? What things might need to change at the policy/government or system/organizational level to improve care for women with disabilities? Probe based on geography (think about limited resource settings) and type of service provider or decision-maker.

14. Is there anything else you would like to add?

Thank you for sharing your experience with us!

Table S1: Participant characteristics (*n* = 31)

Characteristic	No. (%)
**Current primary role**	
Health care or social service provider (e.g., physician, midwife)*	20 (64.5)
Decision-maker (e.g., policy representative, organizational leader, researcher)	11 (35.5)
**Professional designation and/or highest degree†**	
Medical Doctor (MD) (e.g., obstetrician-gynecologist, maternal-fetal medicine, family/primary care physician)	7 (22.6)
Registered Nurse (RN) (e.g., antenatal, public health)	7 (22.6)
Nurse Practitioner (NP)/Registered Nurse-Extended Class (RN-EC)	2 (6.5)
Registered Midwife (RM)	3 (9.7)
Registered Social Worker (RSW, MSW)	6 (19.4)
Occupational Therapy (OT)	2 (6.5)
Graduate degree (e.g., MBA, MPH, MHSc, MScN, MSc, MA, PhD)	13 (41.9)
**Location**	
Large city (population over 1 million)	22 (70.9)
Big city (population over 500,000)	4 (12.9)
Medium-sized city (population over 100,000)	3 (9.7)
Rural/small town (population less than 5,000)	2 (6.5)
**Years in current role**	
Less than 3 years	6 (19.4)
3-5 years	4 (12.9)
6-10 years	6 (19.4)
11-20 years	11 (35.5)
21+ years	3 (9.7)
Missing	1 (3.2)
**Age**	
26-35	3 (9.7)
36-45	12 (38.7)
46-55	8 (25.8)
56-65	5 (16.1)
65+	1 (3.2)
Missing	2 (6.5)
**Gender**	
Women	27 (87.1)
Men	3 (9.7)
Prefer not to answer	1 (3.2)
**Has a disability**	
Yes	7 (22.6)
No	23 (74.2)
Prefer not to answer	1 (3.2)

Notes:
*One midwife was not practicing (on leave) at the time of their interview; one physician was not practicing at the time of their interview.
†Some participants reported more than one designation or degree so total adds up to more than 31.
